# Detection of Early Myocardial Dysfunction by Imaging Biomarkers in Cancer Patients Undergoing Photon Beam vs. Proton Beam Radiotherapy: A Prospective Study

**DOI:** 10.3390/jcdd10100418

**Published:** 2023-10-04

**Authors:** Muhannad Aboud Abbasi, Giulia Bruno, Cristina Di Stefano, Laura Garcia Bello, Nadia N. Laack, Kimberly S. Corbin, Thomas J. Whitaker, Patricia A. Pellikka, Robert W. Mutter, Hector R. Villarraga

**Affiliations:** 1 Department of Cardiovascular Medicine Mayo Clinic, Rochester, MN 55905, USA; 2 Hypertension Unit, Department of Medical Sciences, Città della Salute e della Scienza, University of Torino, 3-10126 Torino, Italy; 3 Department of Radiation Oncology, Mayo Clinic, Rochester, MN 55905, USA; 4 Department of Pharmacology, Mayo Clinic, Rochester, MN 55905, USA

**Keywords:** early cardiotoxicity, radiotherapy, strain rate imaging, thoracic cancer

## Abstract

**1. Background:** We sought to determine acute and subacute changes in cardiac function after proton beam (PBT) and photon beam (PhT) radiotherapy (RT) using conventional and two-dimensional speckle tracking echocardiography (2D-STE) in patients with malignant breast and thoracic tumors. **2. Methods:** Between March 2016 and March 2017, 70 patients with breast or thoracic cancer were prospectively enrolled and underwent transthoracic echocardiography with comprehensive strain analysis at pretreatment, mid-treatment, end of treatment, and 3 months after RT. **3. Results:** PBT was used to treat 44 patients; PhT 26 patients. Mean ± SD age was 55 ± 12 years; most patients (93%) were women. The median (interquartile range) of the mean heart dose was lower in the PBT than the PhT group (47 [27–79] vs. 217 [120–596] cGy, respectively; *p* < 0.001). Ejection fraction did not change in either group. Only the PhT group had reduced systolic tissue Doppler velocities at 3 months. 2D-STE showed changes in endocardial and epicardial longitudinal, radial, and circumferential early diastolic strain rate (SRe) in patients undergoing PhT (global longitudinal SRe, pretreatment vs. end of treatment (*p* = 0.04); global circumferential SRe, pretreatment vs. at 3-month follow-up (*p* = 0.003); global radial SRe, pretreatment vs. at 3-month follow-up (*p* = 0.02) for endocardial values). Epicardial strain values decreased significantly only in patients treated with PhT. Patients in the PhT group had a significant decrease in epicardial global longitudinal systolic strain rate (GLSRs) (epicardial GLSRs, at baseline vs. at end of treatment [*p* = 0.009]) and in GCSRe and GRSRe (epicardial GCSRe, at baseline vs. at 3-month follow-up (*p* = 0.02); epicardial GRSRe, at baseline vs. at 3-month follow-up (*p* = 0.03)) during treatment and follow-up. No changes on 2D-STE were detected in the PBT group. **4. Conclusions:** Patients who underwent PhT but not PBT had reduced tissue Doppler velocities and SRe values during follow-up, suggesting early myocardial relaxation abnormalities. PBT shows promise as a cardiac-sparing RT technology.

## 1. Introduction

Radiotherapy (RT) is an important therapeutic modality for patients with malignant breast and thoracic tumors [1]. Microvascular and macrovascular injuries are the primary pathophysiologic mechanisms of RT-induced myocardial organ damage and described mainly with photon beam therapy ((PhT), i.e., conventional RT). Microvascular dysfunction can lead to an inflammatory response, with subsequent ischemia, fibrosis, and apoptosis. These tissue alterations may decrease myocardial relaxation and contractility, causing diastolic and systolic dysfunction [2].

The risk of radiation-induced heart disease correlates with cardiac dose and volume irradiated, and recent studies have suggested that no safe dose threshold exists under which the risk of cardiac toxicity completely disappears [2,3,4]. Therefore, there is strong rationale for development of new technologies that improve RT delivery. Proton beam therapy (PBT) is one such technology being used clinically for breast, thoracic, and other malignancies [5,6,7]. The normal tissue exposure from PBT differs from PhT because of their distinct physical properties. Protons travel through tissues with minimal dose deposition until they reach the target organ. There, most of the energy is deposited rapidly (Bragg peak) with only minimal deposition occurring in normal tissues beyond the target. In contrast, peak PhT energy deposition is near the entrance into tissues, and the energy deposited slowly decreases with depth, resulting in greater dose deposition to surrounding normal tissues [6,8,9].

Two-dimensional (2D) echocardiography with global longitudinal strain (GLS-basic strain) measurements are currently recommended to assess ventricular function in patients undergoing cancer treatment [10]. Two-dimensional speckle tracking echocardiography (2D-STE) can provide information beyond basic strain (GLS) and includes circumferential, radial, and longitudinal SRs and SRe as well as global circumferential strain (GCS) and global radial strain (GRS) which will be referred to as comprehensive strain analysis. This technique allows for separate measurements of the endocardial and epicardial sheet of the ventricular wall [11,12]. Strain rate during early diastole (SRe) has been shown to identify patients with reduced elastic recoil and impaired relaxation [13]. Outside the realm of cardio-oncology, SRe has demonstrated good sensitivity and specificity in the detection of coronary artery disease, with prognostic utility as well [14], E/SRe was found to be superior to E/e’ ratio in prognosticating outcomes in patients with severe aortic stenosis undergoing aortic valve replacement [15] and in predicting cardiovascular events in patients with myocardial infarction [16]. Stoodley et al. [17] showed that breast cancer patients treated with anthracyclines had reduced SRe even if LVEF was normal. Moreover, a correlation existed between SRe and strain after chemotherapy, confirming that altered diastolic function may indicate compromised systolic function even if it has not yet been detected

The aim of this study was to determine if acute and subacute changes in myocardial mechanics occur in cancer patients treated with PBT or PhT by using both conventional echocardiography and endocardial and epicardial basic and comprehensive 2D-STE.

## 2. Methods

From 1 March 2016 to 1 March 2017, 72 patients who were scheduled for PBT or PhT in the Department of Radiation Oncology at Mayo Clinic in Rochester, Minnesota were prospectively enrolled. TTE examinations were performed at baseline (pretreatment) before the first RT fraction, at mid-treatment, at the end of all scheduled RT sessions, and at 3-month follow-up after completed RT. The study protocol was approved by the Mayo Clinic Institutional Review Board, and written consent forms were signed by all participants.

### 2.1. Inclusion and Exclusion Criteria

**Figure 1:**
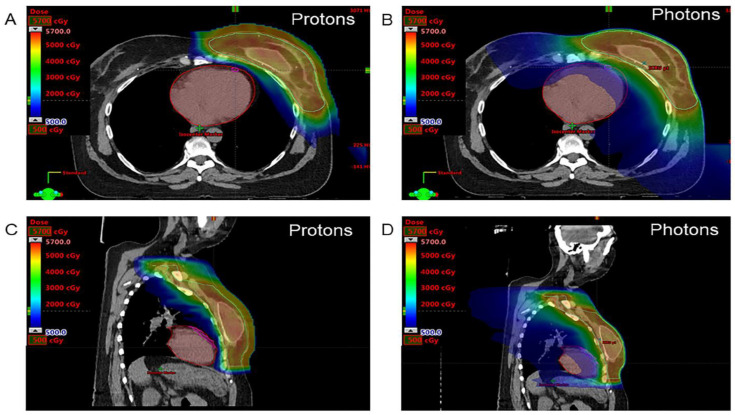
Axial (**A**,**B**) and saggital (**C**,**D**) 500 cGy color-wash images. The images show proton (**A**,**C**) and photon (**B**,**D**) radiotherapy plans at the level of the left ventricle and left anterior descending artery in a woman undergoing postmastectomy radiotherapy for locally advanced left breast cancer. The clinical target volume (cyan), heart (red), and left anterior descending artery (pink) are contoured.

Adult patients were included if they had a diagnosis of breast or thoracic cancer (lung or esophageal cancer) and were scheduled to undergo PBT or PhT with curative intent at an anticipated mean heart radiation dose ≥ 100 cGy, based on a PhT plan. The decision to utilize PBT versus PhT was at the discretion of the patient and treating radiation oncologist based on patient and tumor characteristics and insurance coverage for PBT. Patients treated with prior chemotherapy and those scheduled to undergo concurrent chemoradiotherapy were also included, as were patients treated on a randomized trial comparing conventional and hypofractionated proton postmastectomy radiotherapy [18]. The exclusion criteria were history of cardiovascular diseases (myocardial infarction, congestive heart failure, cardiomyopathy), moderate or severe valvular heart disease, uncontrolled arterial hypertension, cardiac arrhythmias (including atrial fibrillation), and previous thoracic RT. Two patients were excluded from the analysis: 1 who never started RT and 1 who received both PBT and PhT. Therefore, 70 patients were included, and 54 patients had 3-month follow-up results. All patients undergoing PBT were treated with pencil-beam scanning. Patients undergoing PhT were treated with 3-dimensional conformal RT or intensity-modulated RT at physician discretion. All doses for the PhT and PBT groups were prescribed and reported in cGy (relative biological effectiveness, 1.1 × physical dose for PBT [19]). For breast cancer patients, key planning constraint for the heart was mean heart dose ≤ 400 cGy for the PhT group and 75 cGy for the PBT group. For lung cancer, the heart constraint was mean heart dose ≤ 20 Gy for both groups (Figure 1).

### 2.2. 2D Transthoracic Echocardiography

Standard 2D TTE images were acquired with commercially available ultrasound machines (Vivid E95, GE Healthcare Ultrasound System; iE33, Philips Medical Systems Technologies) equipped with a sector probe (M5Sc and S5-1 transducers, respectively). Conventional parameters were assessed according to current guidelines [20]. Left ventricular (LV) diameters and wall thicknesses were measured in parasternal long-axis view. LV geometry was defined by calculating LV mass (using the Devereux formula indexed to body surface area) and relative wall thickness (obtained by dividing double the LV inferolateral wall thickness by the LV internal diameter at end diastole). LV systolic function was assessed by calculating LVEF after estimating LV volumes with the Simpson biplane technique indexed to body surface area. Tissue Doppler systolic velocities measured at the levels of the septal and lateral mitral annulus (s′) were used to study systolic function. LV diastolic function was defined through the evaluation of early diastolic tissue Doppler velocities (e′ waves) of the septal and lateral mitral annulus, mitral valve inflow (pulsed Doppler E- and A-wave velocity), left atrial volume (calculated with the Simpson biplane formula indexed to body surface area), and tricuspid regurgitation peak velocity, according to the current recommendations of the American Society of Echocardiography [21]. LV diastolic filling pressures were estimated by using the ratio of the mitral valve inflow E velocity and the averaged e′ tissue Doppler velocities at the mitral annulus.

### 2.3. 2D Speckle Tracking Echocardiography

Speckle tracking echocardiographic analysis was performed according to current recommendations [22]. Care was taken to optimize frame rates between 40 and 90 frames/second at the time of image acquisition. Standard 2D LV images in apical view (4-, 3-, and 2-chamber views) and parasternal short-axis view at the mid-papillary level were acquired and stored to be analyzed offline (Image Arena Version 4.6 software (TomTec Imaging Systems)). The endocardial borders were traced at end-systole. LV epicardial borders were generated automatically after the endocardial ones were traced. Endocardial and epicardial contours defined the myocardial regions of interest: If needed, the contours could be manually edited, and care was taken to include at least 90% of the myocardium.

GLS, GCS, and GRS as well as longitudinal, circumferential, and radial SRs and SRe were assessed. LV GLS was measured according to the 16-segment model [12]; LV GCS and GRS as well as SRs and SRe were obtained by averaging 6 segments at the parasternal mid short-axis views. Images of more than 2 segments with low-quality tracking were excluded from the final analysis. Results for both endocardial and epicardial strain are reported.

### 2.4. Statistical Analysis

Shapiro–Wilk test was used to determine whether data were normally distributed. Quantitative variables were expressed as mean ± SD or median (interquartile range). Qualitative variables were expressed as frequency and proportion. Between-group comparisons were analyzed with the Student *t* test or Wilcoxon rank sum test for quantitative variables and with χ^2^ or Fisher exact tests for qualitative variables, as appropriate. The restricted maximum likelihood method was used to compare continuous variables between 3 or more paired groups. A *p* value of < 0.05 was assumed as the level of statistical significance. 99% power calculations were run for strain (to detect a 2% difference with a 1.5% SD) and the suggested minimum sample size was 13, and for strain rate (to detect a 0.2 s^−1^ difference with a SD of 0.1 s^−1^) it was 7. Statistical analyses were performed on the entire cohort with JMP version 10.0.0 software (SAS Institute Inc. Cary NC USA). In addition, we separately analyzed the subgroup of patients with breast cancer. For reproducibility, 20 patients were randomly selected, and all echocardiographic parameters were analyzed by 2 independent observers. Reproducibility was calculated by intraclass correlation (α value).

## 3. Results

### 3.1. Study Population

Patients’ clinical characteristics are shown in Table 1. Mean ± SD age was 55.1 ± 11.5 years, and 65 (93%) patients were women. PBT was used in 44 patients, and PhT 26. Breast cancer was the most common diagnosis in both groups. Six patients with lung cancer had PhT (Table 1). There were no significant differences in patient age, sex, cardiovascular risk factors, tumor laterality, and prior or concurrent chemotherapy between the PhT and PBT groups. The treatment techniques have been previously described [23,24]. The median prescription dose (cGy) of the overall cohort was 5000 (4500–5489), PhT 5005 (5000–6000) and PBT 5000 (4005–5000) *p* value < 0.001. The daily dose (cGy) of the overall cohort was 200 (200–260), PhT 200 (200–200) and PBT 200 (200–267) *p* value < 0.003. The median number of fractions of the overall cohort was 25 (15–25), PhT 27 (25–30) PBT 25 (15–25) *p* value < 0.001.

The median mean heart dose (cGy) of the overall cohort was 80 (38–181).

**Figure 2:**
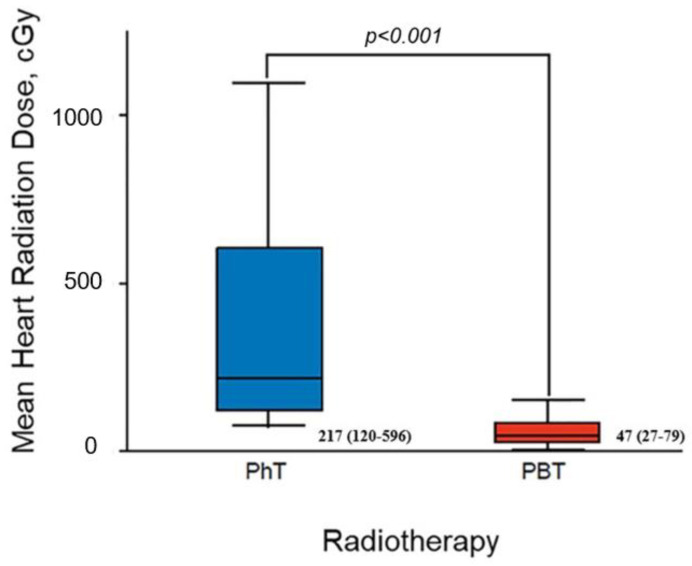
Mean heart radiation dose for patients treated with PhT vs. PBT. The mean dose is expressed as median (interquartile range). PBT indicates proton beam therapy; PhT, photon therapy.

The median of the mean heart dose was significantly lower in the PBT than the PhT group (47 cGy [27–79 cGy] vs. 217 cGy [120–596 cGy]; *p* < 0.001 [Figure 2]). The median mean heart dose was also significantly lower with PBT than PhT in the subset of patients with breast cancer treated (46 cGy [26–77 cGy] vs. 150 cGy [109–231 cGy]; *p* < 0.001).

### 3.2. Conventional Echocardiography

**Figure 3:**
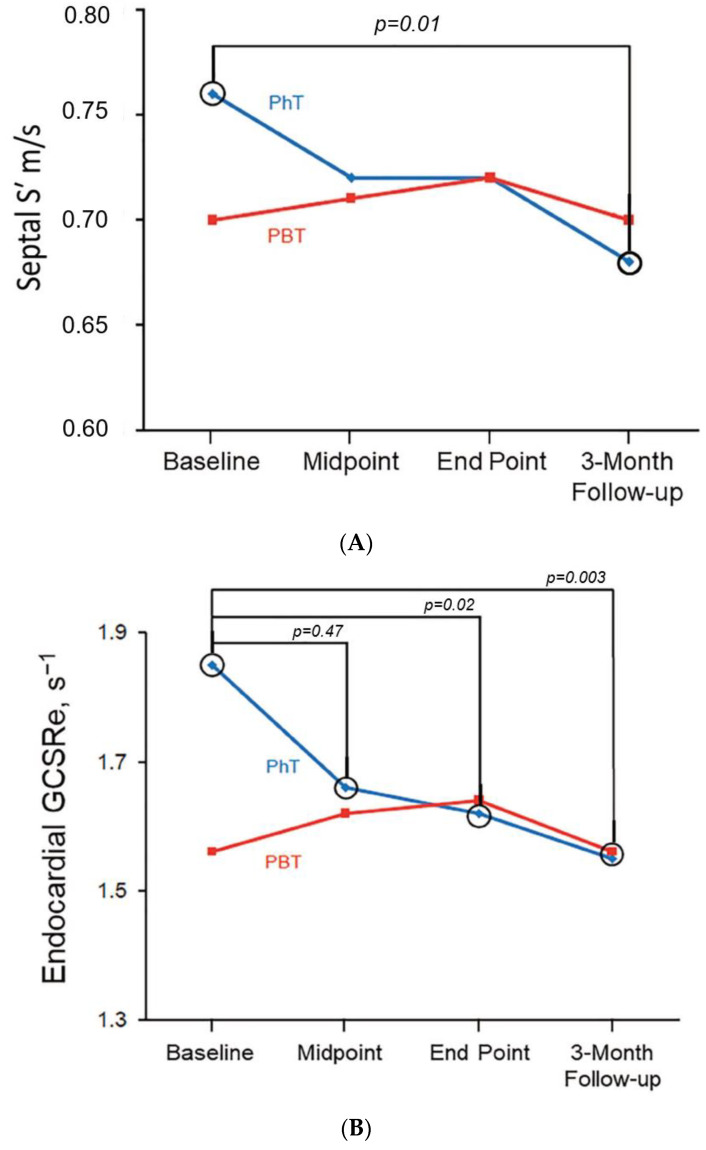
Changes in indicators of cardiac function during treatment. (**A**) Changes in systolic tissue Doppler velocity for patients treated with PhT vs. PBT. (**B**) Changes in GCSRe for patients treated with PhT vs. PBT. GCSRe indicates global circumferential early diastolic strain rate; PBT, proton beam therapy; PhT, photon beam therapy; S′, systolic mitral annular tissue velocity.

Table 2 summarizes all conventional echocardiographic measurements at baseline. At baseline, all parameters of systolic and diastolic function were within normal limits, and there were no differences between the PhT and PBT groups. LV volumes and LVEF did not change throughout RT in either group (Table 3). However, a significant progressive reduction in both LV septal and lateral Doppler systolic velocity (S′) was detected in patients treated with PhT and not with PBT. During PhT, septal S′ decreased from 0.076 ± 0.014 m/s at baseline to 0.068 ± 0.011 m/s at 3-month follow-up TTE (*p* = 0.01; Figure 3A), and lateral S′ declined from 0.077 ± 0.018 m/s at baseline to 0.069 ± 0.016 m/s at 3-month follow-up (*p* = 0.045); the PhT group was also characterized by a transitory reduction in septal e′ from baseline to mid-treatment (*p* = 0.01), followed by a quick recovery after completion of PhT and at 3-month follow-up. In contrast, no significant changes in e’ were detected for patients undergoing PBT (Table 3).

Moreover, when the breast cancer subgroup was analyzed separately, similar alterations in diastolic parameters were found for the PhT group but not for the PBT group. In the PhT group, septal e′ tissue Doppler velocity decreased from 0.083 ± 0.021 m/s at baseline TTE to 0.072 ± 0.019 m/s at mid-treatment TTE (*p* = 0.01), whereas no significant changes appeared during or following PBT. Furthermore, although systolic parameters did not significantly change over the course of breast cancer RT in either group, a nonsignificant tendency in reduction of septal systolic tissue Doppler velocity was detected for patients receiving PhT (from 0.075 ± 0.013 m/s at baseline TTE to 0.069 ± 0.011 m/s at end point TTE (*p* = 0.09)).

### 3.3. 2D Speckle Tracking Echocardiography

Table 4 and Table 5 show results from 2D-STE. Baseline 2D-STE values were within normal limits, and no significant differences were found between the PhT and PBT groups, except in global circumferential early diastolic strain rate (GCSRe) (1.85 ± 0.39 s^−1^ vs. 1.56 ± 0.45 s^−1^, respectively (*p* = 0.006)). No changes in global longitudinal, circumferential, radial strain, or SRs were detected in either the PhT or PBT groups throughout RT and at 3-month follow-up. However, a significant progressive decrease in SRe occurred in patients receiving PhT during treatment. Although global longitudinal early diastolic strain rate (GLSRe) declined significantly during PhT, it improved by the 3-month follow-up visit. In contrast, GCSRe and global radial early diastolic strain rate (GRSRe) worsened throughout RT and during early follow-up (GCSRe, 1.85 ± 0.39 s^−1^ at baseline vs. 1.55 ± 0.36 s^−1^ at 3-month follow-up (*p* = 0.003); GRSRe −2.78 ± 0.96 s^−1^ at baseline vs. −2.23 ± 0.86 s^−1^ at 3-month follow-up (*p* = 0.02, Figure 3B)).

Epicardial strain values decreased significantly only in patients treated with PhT. Patients in the PhT group had a significant decrease in epicardial GLSRs−0.88 ± 0.12 s^−1^ at baseline vs. −0.79 ± 0.13 s^−1^ at end of treatment (*p* = 0.009) and in epicardial GCSRe, 1.01 ± 0.35 s^−1^ at baseline vs. 0.82 ± 0.24 s^−1^ at 3-month follow-up (*p* = 0.02) and epicardial GRSRe, −2.76 ± 0.95 s^−1^ at baseline vs. −2.16 ± 0.87 s^−1^ at 3-month follow-up (*p* = 0.03) during treatment and follow-up.

For the subset of patients treated for breast cancer, no alterations in LV global strain and SRe or SRs values were found in the PBT group throughout RT and follow-up, whereas the PhT group experienced a significant decrease in GLSRe (1.23 ± 0.26 s^−1^ at baseline vs. 1.10 ± 0.27 s^−1^ at end of treatment (*p* = 0.03)). Epicardial SRs values were similarly reduced in the PhT group GLSRs, −0.89 ± 0.12 s^−1^ at baseline vs. −0.76 ± 0.12 s^−1^ at the end of treatment (*p* < 0.001) and GRSRs, 2.70 ± 0.81 s^−1^ at baseline vs. 2.50 ± 0.55 s^−1^ at the end of treatment (*p* = 0.003).

### 3.4. Intraobserver and Interobserver Variability

The analyses showed low intra-observer and inter-observer variability values in tissue Doppler imaging measurements. Correlation coefficients of intra-observer analysis were R^2^ = 0.90 (*p* < 0.001) for septal S′, R^2^ = 0.95 (*p* < 0.001) for lateral S′, R^2^ = 1.0 (*p* < 0.001) for septal e′, and R^2^ = 0.99 (*p* < 0.001) for lateral e′. Correlation coefficients of interobserver analysis were R^2^ = 0.88 (*p* = 0.001) for septal S′, R^2^ = 0.73 (*p* = 0.02) for lateral S′, R^2^ = 0.96 (*p* < 0.001) for septal e′, and R^2^ = 0.92 (*p* < 0.001) for lateral e′.

2D-STE intra-observer correlation coefficients werefor GLS (R^2^ = 0.88; *p* = 0.001), GCS (R^2^ = 0.93; *p* < 0.001), GRS (R^2^ = 0.68; *p* = 0.03), GLSRs (R^2^ = 0.78; *p* = 0.008), GLSRe (R^2^ = 0.65; *p* = 0.04), GCSRe (R^2^ = 0.63; *p* = 0.05), GRSRe (R^2^ = 0.75; *p* = 0.01), epicardial GLS (R^2^ = 0.72; *p* = 0.02), epicardial GCS (R^2^ = 0.78; *p* = 0.02), epicardial GRS (R^2^ = 0.84; *p* = 0.09), epicardial GLSRs (R^2^ = 0.66; *p* = 0.04), epicardial GCSRe (R^2^ = 0.71; *p* = 0.05), and epicardial GRSRe (R^2^ = 0.74; *p* = 0.03). Inter-observer variability correlation coefficients were for GLS (R^2^ = 0.87; *p* = 0.01), GLSRe (R^2^ = 0.84; *p* = 0.002), GRSRe (R^2^ = 0.82; *p* = 0.004), epicardial GLS (R^2^ = 0.84; *p* = 0.004), epicardial GRSRs (R^2^ = 0.80; *p* = 0.009), and epicardial GRSRe (R^2^ = 0.72; *p* = 0.03), whereas there was no significant inter-observer correlation between the other strain measurements.

## 4. Discussion

To our knowledge this is the most comprehensive study of myocardial mechanics performed in patients undergoing modern PBT or PhT. Our main findings were (1) early differences in endocardial and epicardial SRe during RT up to 3 months follow-up in the PhT group, possibly related to early changes in the relaxation properties of the ventricle; (2) normal myocardial mechanics in patients who underwent PBT; and (3) normal LVEF during the entire period evaluated.

### 4.1. Conventional Echocardiography

In our study in the overall cohort, LV volumes and LVEF did not change, but a slight decrease in systolic tissue Doppler velocities occurred in the PhT group during and at 3 months after completion of PhT. Results for diastolic parameters showed reduced septal e′ tissue Doppler velocity in the PhT group at midtreatment, but it improved at the end of RT and during follow-up. Breast cancer patients in the PhT group also had reduced septal e′ tissue Doppler velocity. Our findings are consistent with those of previous studies of cancer patients undergoing PhT [25,26,27]. Ikaheimo et al. [25] showed a transient depression in LV function in a group of breast cancer patients who had undergone adjuvant PhT, with normalization of all parameters within 6 months after RT. Erven and colleagues [28] obtained a similar result for patients with left-sided breast cancer. In that study, LVEF decreased significantly at the end of RT compared with baseline conditions, although values remained within normal limits and recovered within 6 months after completion of treatment. Conversely, another study did not report any changes in LVEF or in other systolic or diastolic parameters in breast cancer patients 12 months from the end of RT [26]. However, another study conducted by Trivedi et al. did report stable LVEF at 6 weeks and 12 months, along with a decreasing E/A ratio indicating potential diastolic impairment [27]. Clasen et al. also reported modest but not statistically significant reduction in ejection fraction per 30-day interval for every 100 cGy increase of mean heart dose in the cohort followed up at 6 weeks and then up to 9 months and no changes in diastolic function [29]. Conventional echocardiographic parameters did not change in patients who underwent PBT during treatment or at 3 months of follow-up, which is the uniqueness of our study comparing both RT groups.

### 4.2. 2D Speckle Tracking Echocardiography

In our study, endocardial GLS, GCS, GRS strain, and SRs did not change in the overall cohort, although endocardial and epicardial SRe gradually decreased during and after PhT, while the PBT group had no changes (Figure 3B and Table 5). Our results were similar when we limited our analysis to the subset of patients undergoing adjuvant RT for breast cancer only. The superiority of 2D-STE imaging in detecting RT-related early myocardial dysfunction changes compared with conventional echocardiography was first noted in a 2007 case report [28]. Erven et al. [26] also showed that in patients with left-sided breast cancer, GLS, and GLSRs decreased after PhT, and both parameters remained significantly lower than baseline conditions within 8 and 14 months from the end of treatment. The same group [28] also reported altered GLS in patients with left-sided breast cancer, but not GLSRs, during PhT and within 2 months after PhT treatment ended. A more recent study [30] focused on patients with left-sided breast cancer who never received chemotherapy and reported impaired deformation parameters at the end of PhT and within 6 weeks of the completion of RT, especially in GLS, SRs, SRe, and radial strain [30]. Another prospective study conducted by Trivedi et al. examining patients diagnosed with left sided breast cancer demonstrated deterioration in strain percentage at 6 weeks postradiotherapy that persisted at 12 months. Interestingly, this was in a dose–response relationship with radiotherapy dose received. (At 6 weeks, myocardial strain was mostly reduced in the anterior wall, followed by the anteroseptal and anterolateral left ventricular walls, and changes in strain persisted at 12 months.) [27] Finally, a study accounting for confounding variables (preexisting cardiovascular disease and an interaction with anthracycline or trastuzumab exposure) reported a modest worsening of GLS per 30-day interval for each 100 cGy increase in MHD, with no changes in circumferential strain

In our cohort, we observed epicardial and endocardial SRe changes only in the PhT group, without significant alterations in GLS, GCS, GRS, and SRs. Changes in SRe may precede alterations in strain and SRs, which may explain why GLS, GCS, GRS, and SRs following PhT did not change. SRe parameters are representative of diastolic function and relate closely to myocardial relaxation and stiffness properties. Relaxation is 1 of 2 processes determined by the active cyclic interaction of myofilaments (contraction follows relaxation and characterizes systole). Myocardial stiffness is dependent not only on myocardial cell features but also on the interstitial matrix (especially related to the grade of fibrosis) [21,31]. We speculate the PhT caused impaired myocardial relaxation during treatment, which manifested with abnormal values in early diastolic strain rate for the three domains of relaxation. These strain rate changes occurred in patients undergoing PhT. 2D-STE is not as angle-dependent as tissue Doppler imaging, allowing regional myocardial function to be detected without being affected by tethering to adjacent segments and overall cardiac motion [1]. Furthermore, early diastolic relaxation is energy-dependent and begins at the LV base and propagates toward the apex, which promotes ventricular relaxation that, along with untwisting and myocardial thinning due to its incompressible nature, results in chamber enlargement, and thus, TDI-based assessment of early relaxation using e’ obtained in the basal medial and lateral annulus may not accurately reflect regional alterations in relaxation or alterations in other segments. As strain rate during early diastole estimated via speckle tracking reflects the sum of all gradients, it is likely that SRe identifies patients with reduced elastic recoil and impaired relaxation [13]. Outside the realm of cardio-oncology, early diastolic strain has demonstrated good sensitivity and specificity in the detection of coronary artery disease, with prognostic utility as well [14]; E/SRe was found to be superior to E/e’ ratio in prognosticating outcomes in patients with severe aortic stenosis undergoing aortic valve replacement [15] and in predicting cardiovascular events in patients with myocardial infarction [16]. Stoodley et al. [17] showed that breast cancer patients treated with anthracyclines had reduced SRe even if LVEF was normal, and in a subgroup with reduced LVEF (<55%) after chemotherapy, GLS values were reduced as well, along with impaired conventional diastolic parameters, E/A ratio, atrial fraction, pulmonary vein diastolic velocity time integral, and A reversal duration. Moreover, a correlation was found between SRe and strain after chemotherapy, confirming that altered diastolic function may indicate compromised systolic function even if it has not yet been detected.

Finally, all patients in our study received state-of-the-art PhT in a specialized practice with techniques such as deep inspiration breath hold to minimize cardiac exposure, which resulted in lower cardiac doses in the PhT group [32,33]. In a recent systematic review of reports published between 2010 and 2015 describing patients receiving RT for breast cancer, the average mean heart dose was 440 cGy, compared with 150 cGy in our study [34]. The relatively low cardiac dose from PhT in our study is likely the primary reason for the reduced rate of cardiac toxicity detected in our PhT-treated patients. Nevertheless, it is noteworthy that even in the setting of modern PhT delivery in a highly specialized practice, early changes in myocardial relaxation still occurred. In contrast, parameters of patients treated with PBT did not change from baseline. These differences likely resulted from a greater than 4-fold reduction in heart dose achieved with PBT compared with PhT. Of note, uncertainty remains regarding the true biologic effect of PBT on normal tissues at the end of the proton range because of the increase in linear energy transfer at the Bragg peak and distal falloff of the proton track [35]. Underwood and colleagues [36] recently reported more asymptomatic lung-density changes after PBT than PhT despite comparable lung doses, which was probably caused by a higher associated transfer of linear energy by PBT in the lung tissues immediately posterior to the chest wall target. In our study, PBT resulted in exceptionally low cardiac exposure and, reassuringly, no changes in cardiac function after PBT. Our results support further investigation of PBT as a promising cardiac-sparing technology [37].

## 5. Limitations

Our study has several limitations. The study population was relatively small. However, it represents one of the largest cohorts of cancer patients treated with different RT modalities who underwent imaging of cardiac function by conventional and 2D-STE to study RT-induced early cardiotoxicity. Furthermore, the patients were not randomized, and therefore, differences in chemotherapy administration, comorbid conditions, insurance status, use of hypofractionation, and other patient or treatment-factor differences between the PBT and PhT groups could have introduced bias. However, the number of patients who received systemic therapy, cardiovascular risk factors, age, and most other clinical factors were balanced between the PBT and PhT groups, and there is no evidence that hypofractionated regimens are more cardioprotective than conventionally fractionated treatments. An additional limitation was the heterogeneity of tumor types in our study. Nevertheless, our results were consistent when we limited the analysis to all patients treated with RT for breast cancer. Finally, short-term follow-up (3 months after completion of RT) may also have underestimated the early cardiotoxicity and regional motion alterations in patients treated with PhT or PBT. Recent studies suggest that radiation-induced major coronary events can occur in the first 5 years after treatment [4,36], and substantial RT-related cardiac organ damage may only appear several years after the end of the treatment [1]. Therefore, further follow-up is needed to understand the long-term impact of PhT and PBT in treating patients with breast and thoracic cancer.

## 6. Conclusions

We have prospectively showed, with 2D-STE, that patients who underwent PhT had reduced endocardial and epicardial circumferential, radial, and longitudinal SRe, which is closely related to myocardial relaxation and is a more sensitive measurement for detecting early cardiac dysfunction than conventional echocardiographic parameters. Patients treated with PBT had no subclinical changes in cardiac function. PBT warrants continued study as a promising cardiac-sparing technology for patients with breast and thoracic cancers.

## Figures and Tables

**Table 1:** General characteristics, cardiovascular risk factors, and oncologic disease ^a^.

Characteristic	Overall Cohort (*n* = 70)	Conventional Photon Therapy (*n* = 26)	Proton Beam Therapy (*n* = 44)	*p* Value
Age, y	55.1 ± 11.5	54.7 ± 11.0	55.3 ± 11.9	0.85
Women (%)	65 (93)	22 (85)	43 (98)	0.06
BMI, kg/m^2^	27.9 ± 5.4	28.4 ± 5.5	27.6 ± 5.4	0.54
BSA, m^2^	1.84 ± 0.23	1.88 ± 0.25	1.82 ± 0.22	0.27
Active smoking (%)	8 (11)	5 (19)	3 (7)	0.24
Obesity (%)	24 (34)	9 (35)	15 (34)	1.0
Hypertension (%)	11 (16)	4 (15)	7 (16)	1.0
Diabetes mellitus (%)	6 (9)	2 (8)	4 (9)	1.0
Dyslipidemia (%)	16 (23)	5 (19)	11 (25)	0.77
Breast/lung/esophageal cancer (%)	62/6/2 (88/9/3)	19/6/1 (73/23/4)	43/0/1 (98/0/2)	0.003
Right/left/bilateral tumor (%)	28/35/7 (40/50/10)	12/13/1 (46/50/4)	16/22/6 (36/50/14)	0.38
Previous systemic therapy (%)	55 (79)	20 (77)	35 (80)	1.0
Concurrent systemic therapy (%)	26 (37)	11 (42)	15 (34)	0.61
Trastuzumab/anthracyclines/taxanes (%)	11/0/5 (16/0/7)	4/0/3 (15/0/12)	7/0/2 (16/0/5)	1.0

Abbreviations: BMI, body mass index; BSA, body surface area. ^a^ All values are No. (%) or mean ± SD.

**Table 2:** Baseline echocardiographic parameters ^a^.

Parameter	Overall Cohort (*n* = 70)	PhT (*n* = 26)	PBT (*n* = 44)	*p* Value
LV mass index, g/m^2^	77.8 ± 16.5	76.8 ± 16.1	78.3 ± 17.0	0.71
RWT	0.41 ± 0.07	0.41 ± 0.07	0.40 ± 0.07	0.45
LVEF, %	61 ± 4	61 ± 4	61 ± 4	0.92
LAVI, mL/m^2^	23.7 ± 7.1	23.2 ± 5.5	24.0 ± 7.9	0.62
E/A	1.08 ± 0.39	1.09 ± 0.46	1.07 ± 0.35	0.80
Septal e′, m/s	0.080 ± 0.023	0.076 ± 0.022	0.082 ± 0.023	0.23
Lateral e′, m/s	0.093 ± 0.029	0.089 ± 0.024	0.095 ± 0.031	0.35
E/e′	8.38 ± 2.99	8.55 ± 3.04	8.29 ± 2.99	0.74
Septal S′, m/s	0.072 ± 0.014	0.076 ± 0.014	0.070 ± 0.012	0.11
Lateral S′, m/s	0.078 ± 0.017	0.077 ± 0.018	0.078 ± 0.017	0.81

Abbreviations: E/A, ratio of early (E) to late (A) transmitral flow velocity; E/e′, ratio between early mitral inflow velocity and mitral annular early diastolic tissue Doppler velocity; e′, early diastolic tissue Doppler velocity; LAVI, left atrial volume index; LV, left ventricular; LVEF, left ventricular ejection fraction; PBT, proton beam therapy; PhT, photon beam therapy. ^a^ All data are expressed as mean ± SD.

**Table 3:** Changes in conventional echocardiographic parameters during treatment and at follow-up for the PhT and PBT Groups ^a^.

	Transthoracic Echocardiography			
Parameter	Baseline	Midpoint	End Point	3-Month Follow-Up
LVEF, %				
PhT	61 ± 4	60 ± 4	61 ± 4	62 ± 3
PBT	61 ± 4	61 ± 4	61 ± 4	61 ± 4
Septal S′, m/s				
PhT	0.076 ± 0.014	0.072 ± 0.015	0.072 ± 0.013	0.068 ± 0.011 ^b^
PBT	0.070 ± 0.012	0.071 ± 0.017	0.072 ± 0.012	0.070 ± 0.015
Lateral S′, m/s				
PhT	0.077 ± 0.018	0.074 ± 0.021	0.080 ± 0.023	0.069 ± 0.016 ^b^
PBT	0.078 ± 0.017	0.079 ± 0.020	0.080 ± 0.022	0.085 ± 0.030
LAVI, mL/m^2^				
PhT	23.2 ± 5.5	22.0 ± 6.1	22.5 ± 6.3	24.2 ± 6.9
PBT	24.0 ± 7.9	25.9 ± 7.8	25.1 ± 9.0	26.6 ± 7.2
E/A				
PhT	1.09 ± 0.46	1.10 ± 0.41	1.06 ± 0.52	1.10 ± 0.43
PBT	1.07 ± 0.35	1.10 ± 0.37	1.08 ± 0.38	1.08 ± 0.35
E/e′				
PhT	8.40 ± 2.95	8.65 ± 2.25	8.40 ± 2.22	8.56 ± 2.43
PBT	8.29 ± 2.99	7.73 ± 2.99	7.76 ± 2.24	7.85 ± 2.41
Septal e′, m/s				
PhT	0.077 ± 0.021	0.069 ± 0.019 ^b^	0.074 ± 0.020	0.079 ± 0.024
PBT	0.082 ± 0.023	0.087 ± 0.025	0.088 ± 0.028	0.090 ± 0.032
Lateral e′, m/s				
PhT	0.089 ± 0.024	0.092 ± 0.031	0.094 ± 0.028	0.097 ± 0.030
PBT	0.095 ± 0.031	0.102 ± 0.032	0.100 ± 0.033	0.099 ± 0.034

Abbreviations: E/A, ratio of early (E) to late (A) mitral inflow velocity; E/e′, ratio between early mitral inflow velocity and mitral annular early diastolic tissue velocity; e′, early diastolic tissue Doppler velocity; LAVI, left atrial volume indexed to body surface area; LVEF, left ventricular ejection fraction; PBT, proton beam therapy; PhT, photon therapy; S′ TDI, systolic tissue Doppler velocity. ^a^ All data expressed as mean ± SD. ^b^ *p* < 0.05 from the baseline value.

**Table 4:** Baseline two-dimensional speckle tracking echocardiographic parameters ^a^.

Parameter	Overall Cohort (*n* = 70)	PhT (*n* = 26)	PBT (*n* = 44)	*p* Value
GLS, %	−18.82 ± 2.53	−18.03 ± 2.78	−19.26 ± 2.28	0.07
GCS, %	−24.19 ± 4.36	−24.99 ± 4.58	−23.70 ± 4.20	0.25
GRS, %	56.93 ± 25.42	55.67 ± 14.49	57.70 ± 30.32	0.71
RV S, %	−20.98 ± 3.52	−20.78 ± 2.84	−21.09 ± 3.88	0.71
RV free wall S, %	−22.86 ± 4.84	−22.32 ± 4.49	−23.16 ± 5.05	0.49
GLSRs, s^−1^	−1.09 ± 0.21	−1.10 ± 0.27	−1.09 ± 0.17	0.88
GCSRs, s^−1^	−1.61 ± 0.40	−1.69 ± 0.44	−1.55 ± 0.37	0.18
GRSRs, s^−1^	2.70 ± 0.80	2.73 ± 0.75	2.67 ± 0.84	0.78
GLSRe, s^−1^	1.14 ± 0.25	1.17 ± 0.25	1.12 ± 0.25	0.43
GCSRe, s^−1^	1.67 ± 0.45	1.85 ± 0.39	1.56 ± 0.45	0.006
GRSRe, s^−1^	−2.50 ± 0.86	−2.78 ± 0.96	−2.33 ± 0.75	0.05

Abbreviations: GCS, global circumferential strain; GCSRs, global circumferential systolic strain rate; GCSRe, global circumferential early diastolic strain rate; GLS, global longitudinal strain; GLSRs, global longitudinal systolic strain rate; GLSRe, global longitudinal early diastolic strain rate; GRS, global radial strain; GRSRs, global radial systolic strain rate; GRSRe, global radial early diastolic strain rate; PBT, proton beam therapy; PhT, photon beam therapy. ^a^ All values are expressed as mean ± SD.

**Table 5:** Changes in 2D-STE parameters during treatment and follow-up in the PhT and PBT groups ^a^.

	Transthoracic Echocardiography			
Parameter	Baseline	Midpoint	End Point	3-Month Follow-Up
GLS, %				
PhT	−18.03 ± 2.78	−18.13 ± 1.99	−17.84 ± 2.60	−19.07 ± 2.32
PBT	−19.26 ± 2.28	−19.33 ± 2.74	−19.41 ± 2.50	−18.95 ± 2.25
GCS, %				
PhT	−24.99 ± 4.58	−25.08 ± 3.26	−23.41 ± 5.14	−24.06 ± 3.92
PBT	−23.70 ± 4.20	−23.60 ± 4.30	−24.45 ± 3.72	−23.83 ± 3.64
GRS, %				
PhT	55.67 ± 14.49	49.04 ± 16.78	54.67 ± 22.91	61.01 ± 20.38
PBT	57.70 ± 30.32	56.18 ± 22.79	61.58 ± 23.20	63.24 ± 17.10
GLSRs, s^−1^				
PhT	−1.10 ± 0.27	−1.11 ± 0.16	−1.09 ± 0.18	−1.11 ± 0.17
PBT	−1.09 ± 0.17	−1.12 ± 0.19	−1.09 ± 0.18	−1.10 ± 0.16
GCSRs, s^−1^				
PhT	−1.69 ± 0.44	−1.66 ± 0.29	−1.56 ± 0.28	−1.61 ± 0.34
PBT	−1.55 ± 0.37	−1.56 ± 0.33	−1.58 ± 0.31	−1.60 ± 0.33
GRSRs, s^−1^				
PhT	2.73 ± 0.75	2.57 ± 0.63	2.57 ± 0.63	2.79 ± 0.79
PBT	2.67 ± 0.84	2.60 ± 0.83	2.74 ± 0.68	2.92 ± 0.70
GLSRe, s^−1^				
PhT	1.17 ± 0.25	1.07 ± 0.22 ^b^	1.06 ± 0.26 ^b^	1.10 ± 0.23
PBT	1.12 ± 0.25	1.06 ± 0.29	1.10 ± 0.27	1.13 ± 0.24
GCSRe, s^−1^				
PhT	1.85 ± 0.39	1.66 ± 0.29 ^b^	1.62 ± 0.31 ^b^	1.55 ± 0.36 ^b^
PBT	1.56 ± 0.45	1.62 ± 0.57	1.64 ± 0.44	1.56 ± 0.29
GRSRe, s^−1^				
PhT	−2.78 ± 0.96	−2.35 ± 0.74 ^b^	−2.68 ± 0.90	−2.23 ± 0.86 ^b^
PBT	−2.33 ± 0.75	−2.28 ± 0.94	−2.38 ± 0.73	−2.48 ± 0.68

Abbreviations: 2D-STE, 2-dimensional speckle tracking echocardiography; GCS, global circumferential strain; GCSRs, global circumferential systolic strain rate; GCSRe, global circumferential early diastolic strain rate; GLS, global longitudinal strain; GLSRs, global longitudinal systolic strain rate; GLSRe, global longitudinal early diastolic strain rate; GRS, global radial strain; GRSRs, global radial systolic strain rate; GRSRe, global radial early diastolic strain rate; PBT, proton beam therapy; PhT, photon beam therapy. ^a^ All values are expressed as mean ± SD. ^b^ *p* < 0.05 from the baseline value.

## Data Availability

All the data within this study are provided in the manuscript.

## Abbreviations

2D	2-dimensional
2D-STE	2-dimensional speckle tracking echocardiography
A	late diastolic mitral inflow velocity
E	early diastolic mitral inflow velocity
e′	early diastolic tissue Doppler velocity
E/A	ratio of early to late diastolic mitral inflow velocities
GCS	global circumferential strain
GCSRe	global circumferential early diastolic strain rate
GLS	global longitudinal strain
GLSRe	global longitudinal early diastolic strain rate
GLSRs	global longitudinal systolic strain rate
GRS	global radial strain
GRSRe	global radial early diastolic strain rate
LV	left ventricular
LVEF	left ventricular ejection fraction
PBT	proton beam therapy
PhT	photon beam therapy
RT	radiotherapy
RV	right ventricular
S′	systolic annular tissue velocity
SRe	early diastolic strain rate
SRs	systolic strain rate
TAPSE	tricuspid annular plane systolic excursion
TTE	transthoracic echocardiography
